# Toward Smart Home Authentication Using PUF and Edge-Computing Paradigm

**DOI:** 10.3390/s22239174

**Published:** 2022-11-25

**Authors:** Tsu-Yang Wu, Fangfang Kong, Liyang Wang, Yeh-Cheng Chen, Saru Kumari, Jeng-Shyang Pan

**Affiliations:** 1 College of Computer Science and Engineering, Shandong University of Science and Technology, Qingdao 266590, China; 2 Department of Computer Science, University of California, Davis, CA 001313, USA; 3 Department of Mathematics, Chaudhary Charan Singh University, Meerut 250004, India; 4 Department of Information Management, Chaoyang University of Technology, Taichung 41349, Taiwan

**Keywords:** IoT, edge computing, smart home, PUF

## Abstract

The smart home is a crucial embodiment of the internet of things (IoT), which can facilitate users to access smart home services anytime and anywhere. Due to the limited resources of cloud computing, it cannot meet users’ real-time needs. Therefore, edge computing emerges as the times require, providing users with better real-time access and storage. The application of edge computing in the smart home environment can enable users to enjoy smart home services. However, users and smart devices communicate through public channels, and malicious attackers may intercept information transmitted through public channels, resulting in user privacy disclosure. Therefore, it is a critical issue to protect the secure communication between users and smart devices in the smart home environment. Furthermore, authentication protocols in smart home environments also have some security challenges. In this paper, we propose an anonymous authentication protocol that applies edge computing to the smart home environment to protect communication security between entities. To protect the security of smart devices, we embed physical unclonable functions (PUF) into each smart device. Real-or-random model, informal security analysis, and ProVerif are adopted to verify the security of our protocol. Finally, we compare our protocol with existing protocols regarding security and performance. The comparison results demonstrate that our protocol has higher security and slightly better performance.

## 1. Introduction

The internet of things (IoT) [1,2,3,4] is a network connected with everything, which can collect various types of information in real time and communicate with other devices. The development of the IoT has brought significant achievements in different fields, such as smart city [5,6,7,8], healthcare [9,10,11], vehicular ad hoc network (VANET) [12,13,14,15,16], smart home [17,18,19], and artificial intelligence [20,21]. The smart home is the embodiment of IoT. It is an environment in which smart devices are deployed in the house, and various devices provide services to users through connecting to the internet. People can access smart home services anytime and anywhere through voice assistants or applications and easily control smart devices. In the smart home environment, people’s live have become more comfortable, their lifestyle has become more intelligent, and people’s quality of life is also constantly improving.

**Figure 1:**
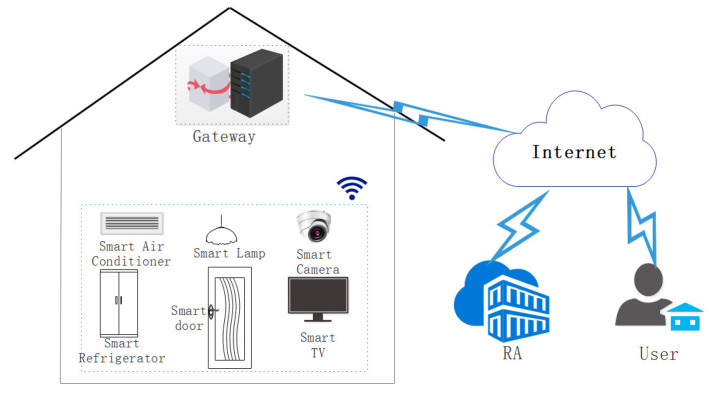
Traditional architecture of smart home.

Many smart devices are deployed in the smart home environment, such as smart air conditioners, smart desk lamps, and smart curtains. These smart devices can provide users with various services. The traditional framework of the smart home is shown in Figure 1. The framework consists of four entities: registration authority (RA), users, gateway, and smart devices. The primary responsibilities of RA include the registration of users and smart devices as well as the distribution of system parameters. Gateway is a bridge between smart devices and users. Only smart devices registered in RA can provide services for users. Users use mobile devices (such as smartphones, tablets, and smartwatches) to control smart devices in their homes at any time. For example, users can turn on the air conditioner and close the curtains outdoors; users can master the family situation by viewing the smart camera.

The traditional smart home architecture relies on centralized cloud computing, which is used for data collection and processing. There are some problems in the traditional architecture; for example, in monitoring this application scenario that requires real-time feedback, cloud computing [22,23,24] will process a great deal of data, which may not meet users’ real-time needs [25,26]. Edge computing [27,28,29] is closer to the data source than cloud computing. It can better process data and provide real-time access, solving the above problems. An edge gateway is the node of edge computing, which can give real-time computing and storage in the smart home environment instead of going to the remote cloud center. The edge gateway can locally process the data collected between the user’s mobile device and the smart device. First, the user and the smart device are registered in the registration center, and the registered legal user negotiates the session key with the smart device with the help of the edge gateway. Only legal users can enjoy smart home services. Although smart homes bring convenience to people’s lives, users and smart devices communicate through public channels. Due to the openness of the public channel, the information transmitted in the public channel may be intercepted by malicious attackers, which will lead to user privacy disclosure. Therefore, protecting users and smart devices for secure communication is very important.

The physical unclonable function (PUF) [30,31] is a function that can be embedded in an integrated circuit. The integrated circuit takes a bit string as input (or called challenge) and generates a random response string as the output. For various PUF modules manufactured on the same integrated circuit, no two PUF modules will produce the same response if faced with the same challenges. If a malicious attacker wants to change or destroy the PUF, it will change the corresponding internal circuit and logic gate delay. At this time, even if the same challenge is entered, the malicious attacker cannot obtain the same response. According to the microstructure and response of a given PUF, it is difficult for a malicious attacker to guess or infer the correct challenge. Moreover, the PUF is available on demand and does not require secure storage.

In this paper, a smart home authentication protocol using PUF and edge computing paradigm is proposed. The following are the novelty and contributions of this paper:

(1) To the best of our knowledge, we are the first to introduce an edge-computing-based smart home architecture and propose an authentication protocol based on this architecture. In our protocol, the user and the smart device realize mutual authentication with the help of the edge gateway and successfully establish a session key for secure communication.
(2) We apply PUF to smart devices to prevent data-leakage attacks launched by attackers, thus ensuring data security. According to the security properties of PUF, even if an attacker gets the same challenge, they cannot get the same response. Therefore, using PUF in our protocol can resist tampering and biological cloning attacks.
(3) We verify the security of our protocol by using the real or random (ROR) model, informal security analysis and simulation software (ProVerif). The results are shown that the proposed protocol can resist several well-known attacks.
(4) Finally, we compare our protocol with existing protocols regarding security and performance. The comparisons demonstrate that our protocol guarantees better security and slightly lower communication cost.

The remainder of this paper is structured as follows. The relevant research on smart homes, edge computing, and PUF is briefly reviewed in Section 2. In Section 3, we describe the system model and detailed protocol. We prove the security of our proposed protocol in Section 4. In Section 5, we compare our protocol with existing protocols in terms of security and performance. In Section 6, we set forth our conclusions.

## 2. Related Work

Many researchers proposed several authentication and key agreement (AKA) protocols in different environments. In 2008, Jeong et al. [32] proposed a lightweight user authentication protocol in the home network environment. This protocol could not guarantee the anonymity of users, and users were easily tracked. In addition, the protocol could not resist attacks by privileged insiders. Vaidya et al. [33] proposed a strong cryptographic-based AKA protocol in the home network environment. The author showed that this protocol has strong security. However, Kim et al. [34] performed cryptanalysis on the protocol of Vaidya et al. [33] and found that their protocol could not provide forward security and suffered from stolen smart card attacks. Kim et al. [34] indicated the security vulnerabilities of Vaidya et al.’s protocol [33] and proposed an enhanced AKA. Unfortunately, the protocol of Kim et al. [34] could not resist privileged insider attacks and was unable to guarantee users’ anonymity and untraceability. In 2017, Wazid et al. [35] proposed a lightweight AKA for remote users. They proved that their protocol was secure and had good performance. However, Lyu et al. [36] discovered that the protocol of Wazid et al. [35] was unable to withstand stolen verifier attacks and synchronization attacks. Lyu et al. [36] introduced IFTTT as a home gateway and used it as the executor and supervisor of commands. In 2019, Shuai et al. [37] proposed an efficient AKA protocol using elliptic curve cryptography (ECC) and showed that the protocol could resist existing attacks. However, Kaur and Kumar [38] found that Shuai et al.’s protocol [37] was vulnerable to insider attacks, replay attacks, and offline password guessing attacks. Subsequently, Kaur and Kumar [38] proposed a two-factor AKA protocol to enhance security. Unfortunately, Yu et al. [18] found that the protocol of Kaur and Kumar [38] could not resist impersonation attacks and session key disclosure attacks and proposed a secure AKA protocol based on three factors. However, Alzahrani et al. [39] discovered that Yu et al.’s protocol [18] was unable to achieve mutual authentication. Banerjee et al. [40] found that Shuai et al.’s protocol [37] could not resist stolen smart card attacks and user impersonation attacks and then proposed an efficient anonymous authentication protocol. Unfortunately, this protocol cannot guarantee the anonymity and untraceability of users [41]. Oh et al. [42] proposed an efficient authentication protocol using the hush function for IoT-based smart home environments. They proved that the protocol can resist known attacks.

In edge-computing environments, Tsai and Lo [43] proposed an authentication protocol using identity-based encryption technology. This protocol is based on bilinear pairing and the identity-based cryptosystem, which reduced the computation of users and servers. However, Jiang et al. [44] proved that the protocol of Tsai and Lo [43] was vulnerable to server impersonation attacks. Irshad et al. [45] also found that Tsai and Lo’s protocol [43] could not resist the de-synchronization attacks. They designed an improved multi-server authentication protocol and proved that the designed protocol could resist known attacks. However, Xiong et al. [46] pointed out that the protocol of Irshad et al. [45] lacked the registration and revocation of users and designed a new protocol. Later, Jia et al. [47] designed an identity-based authentication protocol. However, Li et al. [26] found that Jia et al.’s protocol [47] could not resist man-in-the-middle (MITM) attacks and then proposed a novel mobile edge computing environment architecture and designed a lightweight AKA protocol on this architecture. Unluckily, Li et al.’s protocol [26] cannot resist replay attacks and denial of service attacks. Kaur et al. [48] proposed a lightweight privacy-preserving AKA protocol, which adopts elliptic curve cryptography to resist various attacks, thus ensuring secure communication between entities.

Numerous PUF-based AKA protocols were recently proposed to address the aforementioned well-known security issues. Aysu et al. [49] proposed a secure and efficient end-to-end AKA protocol based on PUF between servers and resource-limited devices. Chatterjee et al. [50] designed a PUF-based AKA protocol for the IoT to realize authentication and secure information transfer between devices. Braeken [51] analyzed Chatterjee’s protocol [50] and found that it could not resist MITM attacks and replay attacks and proposed an efficient AKA protocol. Gope et al. [52] proposed a lightweight AKA protocol for user privacy protection in industrial wireless sensor networks. In this protocol, user and sensor nodes can authenticate and negotiate the session key with the aid of the gateway. Chen et al. [53] found that PUF-authentication protocols are vulnerable to machine learning attacks. Therefore, they adopted the concept of Shamir’s secret sharing to design an AKA protocol to resist the attacks. Ebrahimabadi et al. [54] designed a novel authentication protocol based on PUF and showed that the protocol has better security and efficiency. In order to ensure that users can obtain secure and timely services in a smart city environment, Yu et al. [55] proposed a lightweight authentication protocol based on PUF in an internet of drones environment. Shao et al. [56] proposed an AKA protocol using PUF in a wireless medical sensor environment with limited resources to ensure data security and patient privacy. Some significant relevant works are listed in Table 1.

## 3. Proposed Protocol

**Figure 2:**
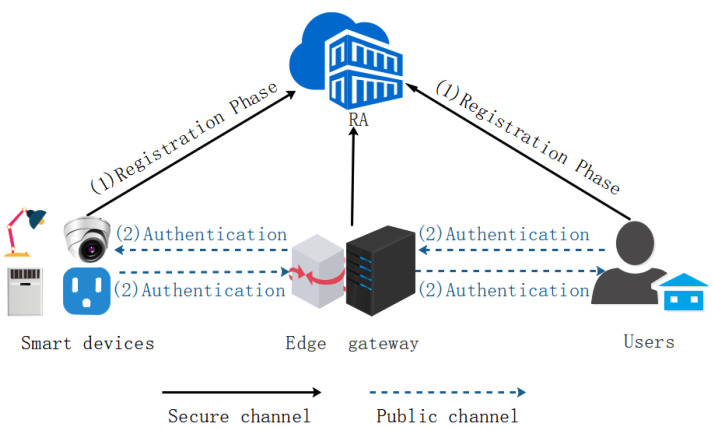
System model.

In this section, an authentication protocol using PUF and the edge-computing paradigm for the smart home environment is proposed. Four entities, trusted third party TTP, edge gateway EGW, user $U_{i}$, and smart device $SD_{j}$, are involved in our protocol. The system model is shown in Figure 2. Details on each entity are described below:

(1) Trusted third party TTP: TTP is a trusted entity, mainly responsible for the registration of home users and smart devices. Additionally, it stores a few users and smart device registration parameters in the edge gateway’s secure database.
(2) Edge gateway EGW: EGW is a trusted entity and is deployed in the home. EGW can collect data from various smart devices, process the data, and send the processed data to users who need data. It also serves as a bridge between smart devices and users.
(3) Home user $U_{i}$: $U_{i}$ refers to the legal users who have successfully registered through TTP. With the help of the EGW, legal home users can enjoy the services provided by smart devices and remotely control them through mobile devices (such as smartphones, tablets, and smartwatches) anytime and anywhere.
(4) Smart device $SD_{j}$: $SD_{j}$ deployed in the smart home environment (such as cameras, smart refrigerators, smart desk lamps, and smart locks) must be registered with TTP. Each smart device is embedded with a PUF module. In the smart home, it can execute the instructions transmitted by the user through the edge gateway and collect the data.

Our protocol is divided into the registration, login and authentication phases. Before $U_{i}$ and $SD_{j}$ are deployed in the smart home environment, TTP generates a master key *x*. Each $SD_{j}$ has a unique identity $SMID_{j}$ and PUF module. The symbols used in the protocol are shown in Table 2, and the following thoroughly explains each phase.

### 3.1. Registration Phase

In the smart home environment, $U_{i}$ and $SD_{j}$ must register with TTP via a secure channel. There are two phases of registration: $U_{i}$ registration and $SD_{j}$ registration.

**Figure 3:**
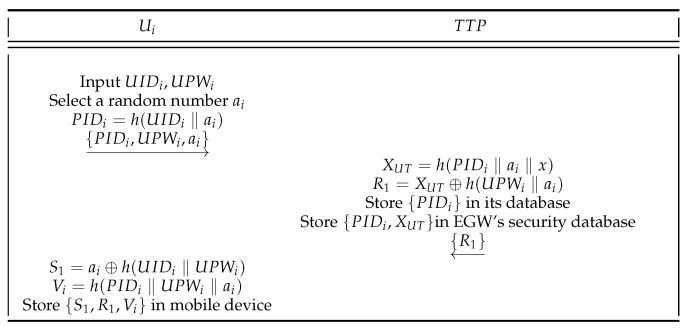
$U_{i}$ registration phase.

**User Registration Phase.** If $U_{i}$ wants to enjoy smart home services, he or she must first register as a legal user in TTP. The process of $U_{i}$ registration is shown in Figure 3. The steps of $U_{i}$ registration are described in detail below.

(1) To begin with, $U_{i}$ uses the mobile device to enter the identity $UID_{i}$, password $UPW_{i}$, and selects a random number $a_{i}$. Then, the mobile device calculates
  (1) $$PID_{i}=h\left(UID_{i}\|a_{i}\right).$$
  Finally, $U_{i}$ passes $\left\{PID_{i},UPW_{i},a_{i}\right\}$ to TTP through the secure channel.
(2) When TTP receives $\left\{PID_{i},UPW_{i},a_{i}\right\}$, first retrieves $PID_{i}$ from the database of TTP. If retrieved in the database, TTP rejects the $U_{i}$’s registration. Or else, TTP calculates
  (2) $$\begin{array}{c} X_{UT}=h(PID_{i}\|a_{i}\left\|x\right),\\ R_{1}=X_{UT}⊕h\left(UPW_{i}\|a_{i}\right). \end{array}$$
  Thereafter, TTP calculation is completed, stores $\left\{PID_{i}\right\}$ in its database, and stores $\left\{PID_{i},X_{UT}\right\}$ in the secure database of EGW. Finally, TTP sends $\left\{R_{1}\right\}$ to $U_{i}$.
(3) When $U_{i}$ receives $\left\{R_{1}\right\}$, it calculates
  (3) $$\begin{array}{c} S_{1}=a_{i}⊕h\left(UID_{i}\|UPW_{i}\right),\\ V_{i}=h(PID_{i}\|RPW_{i}\left\|a_{i}\right). \end{array}$$
  Finally, $U_{i}$ stores $\{S_{1},R_{1},V_{i}\}$ in the mobile device.

**Figure 4:**
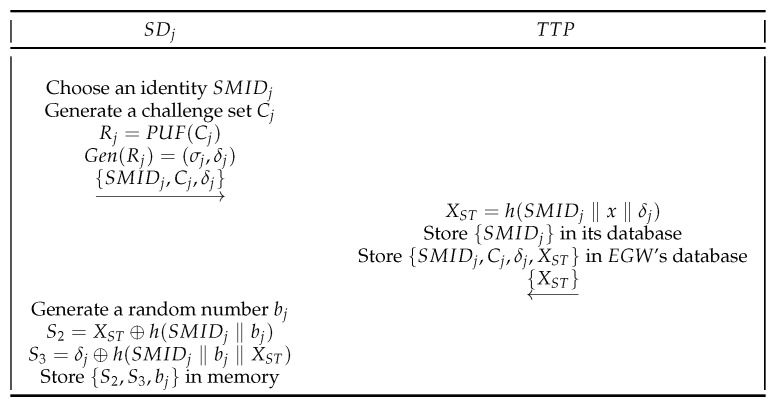
$SD_{j}$ registration phase.

**Smart Device Registration Phase.**$SD_{j}$ must be registered at TTP before it can provide smart home services to $U_{i}$. The $SD_{j}$ registration process is shown in Figure 4. The following are the specific $SD_{j}$ registration process.

(1) Initially, $SD_{j}$ selects an identity $SMID_{j}$, generates a challenge $C_{j}$. Then, $SD_{j}$ calculates
  (4) $$\begin{array}{c} R_{j}=PUF\left(C_{j}\right),\\ Gen\left(R_{j}\right)=\left(\sigma_{j},\delta_{j}\right). \end{array}$$
  Finally, $SD_{j}$ sends $\left\{SMID_{j},C_{j},\delta_{j}\right\}$ to TTP.
(2) After receiving $\left\{SMID_{j},C_{j},\delta_{j}\right\}$, TTP retrieves $SMID_{j}$ from the database. If not retrieved in the database, TTP calculates
  (5) $$X_{ST}=h(SMID_{j}\left\|x\right\|\delta_{j}).$$
  Then, TTP stores $\left\{SMID_{j}\right\}$ in its database and stores $\left\{SMID_{j},C_{j},\delta_{j},X_{ST}\right\}$ in EGW’s security database. Finally, TTP sends $\left\{X_{ST}\right\}$ to $SD_{j}$.
(3) After receiving $\left\{X_{ST}\right\}$, $SD_{j}$ generates a random number $b_{j}$, and then calculates
  (6) $$\begin{array}{c} S_{2}=X_{ST}⊕h\left(SMID_{j}\|b_{j}\right),\\ S_{3}=\delta_{j}⊕h(SMID_{j}\|b_{j}\left\|X_{ST}\right). \end{array}$$
  Finally, $SD_{j}$ stores $\left\{S_{2},S_{3},b_{j}\right\}$ in memory.

### 3.2. Login and Authentication Phase

**Figure 5:**
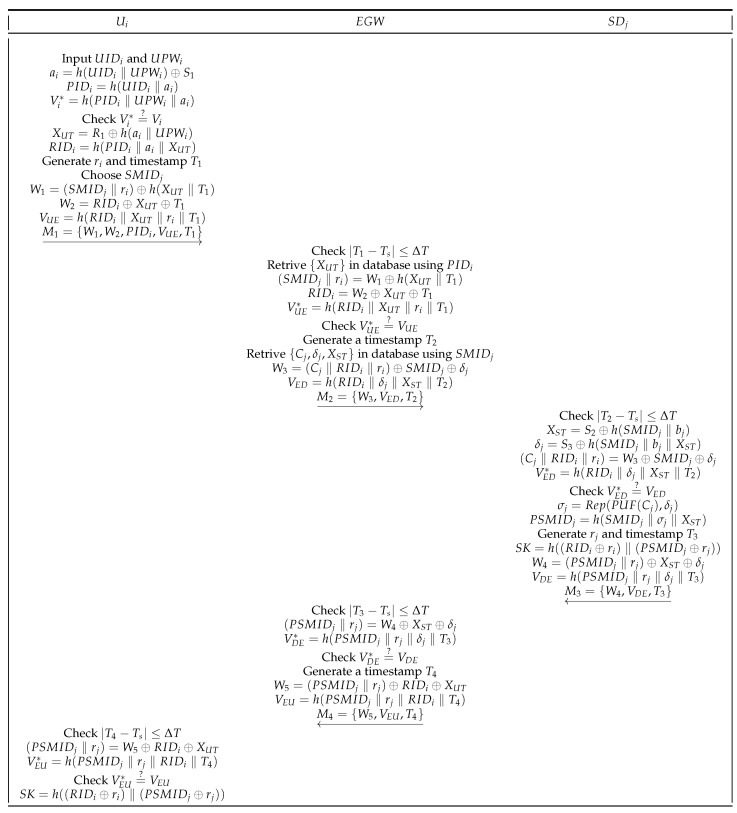
Login and authentication phase.

In this phase, all entities communicate via a public channel. With the help of the EGW, the legal $U_{i}$ establishes a session key SK with the $SD_{j}$. The established SK facilitates the $U_{i}$ to safely obtain the service of the $SD_{j}$ and future communication. The detailed login and authentication process is shown in Figure 5. The steps of this process are described in detail below.

(1) First, $U_{i}$ uses the mobile device to input his own identity $UID_{i}$, password $UPW_{i}$, and then calculates
  (7) $$\begin{array}{c} a_{i}=h\left(UID_{i}\|UPW_{i}\right)⊕S_{1},\\ PID_{i}=h\left(UID_{i}\|a_{i}\right),\\ V_{i}^{*}=h(PID_{i}\|RPW_{i}\left\|a_{i}\right). \end{array}$$
  Next, $U_{i}$ check $V_{i}^{*}\overset{?}{=}V_{i}$. If it holds, $U_{i}$ successfully logs in. Otherwise, $U_{i}$ will be denied login. Then, $U_{i}$ calculates
  (8) $$\begin{array}{c} X_{UT}=R_{1}⊕h\left(a_{i}\|UPW_{i}\right),\\ RID_{i}=h(PID_{i}\|a_{i}\left\|X_{UT}\right). \end{array}$$
  Additionally, $U_{i}$ selects unique identity $SMID_{j}$ of the $SD_{j}$, random number $r_{i}$, and $T_{1}$. Then, $U_{i}$ calculates
  (9) $$\begin{array}{c} W_{1}=\left(SMID_{j}\|r_{i}\right)⊕h\left(X_{UT}\|T_{1}\right),\\ W_{2}=RID_{i}⊕X_{UT}⊕T_{1},\\ V_{UE}=h(RID_{i}\|X_{UT}\|r_{i}\left\|T_{1}\right). \end{array}$$
  At last, $U_{i}$ sends the message $M_{1}=\left\{W_{1},W_{2},PID_{i},V_{UE},T_{1}\right\}$ through the public channel to EGW.
(2) When receiving $M_{1}$ sent by $U_{i}$, EGW first checks $|T_{1}-T_{s}|\leq \Delta T$. If $T_{1}$ is valid, EGW uses $PID_{i}$ retrieve $X_{UT}$ from the secure database, and calculates
  (10) $$\begin{array}{c} \left(SMID_{j}\|r_{i}\right)=W_{1}⊕h\left(X_{UT}\|T_{1}\right),\\ RID_{i}=W_{2}⊕X_{UT}⊕T_{1},\\ V_{UE}^{*}=h(RID_{i}\|X_{UT}\|r_{i}\left\|T_{1}\right). \end{array}$$
  Next, EGW checks $V_{UE}^{*}\overset{?}{=}V_{UE}$. If it is correct, EGW authenticates $SD_{j}$, and uses $SMID_{j}$ retrieves $\left\{C_{j},\delta_{j},X_{ST}\right\}$ from the secure database. Then, EGW generates a timestamp $T_{2}$, and calculates
  (11) $$\begin{array}{c} W_{3}=(C_{j}\|RID_{i}\left\|r_{i}\right)⊕SMID_{j}⊕\delta_{j},\\ V_{ED}=h(RID_{i}\|\delta_{j}\|X_{ST}\left\|T_{2}\right). \end{array}$$
  Eventually, EGW sends the message $M_{2}=\left\{W_{3},V_{ED},T_{2}\right\}$ to $SD_{j}$.
(3) When $SD_{j}$ receives $M_{2}$ sent from EGW, first checks $|T_{2}-T_{s}|\leq \Delta T$. Then, $SD_{j}$ calculates
  (12) $$\begin{array}{c} X_{ST}=S_{2}⊕h\left(SMID_{j}\|b_{j}\right),\\ \delta_{j}=S_{3}⊕h(SMID_{j}\|b_{j}\left\|X_{ST}\right),\\ (C_{j}\|RID_{i}\left\|r_{i}\right)=W_{3}⊕SMID_{j}⊕\delta_{j},\\ V_{ED}^{*}=h(RID_{i}\|\delta_{j}\|X_{ST}\left\|T_{2}\right). \end{array}$$
  Next, $SD_{j}$ checks $V_{ED}^{*}\overset{?}{=}V_{ED}$. If it is correct, the identity of the EGW is authenticated, then $SD_{j}$ calculates
  (13) $$\begin{array}{c} \sigma_{j}=Rep\left(PUF\left(C_{j}\right),\delta_{j}\right),\\ PSMID_{j}=h(SMID_{j}\|\sigma_{j}\left\|X_{ST}\right). \end{array}$$
  Additionally, $SD_{j}$ generates $r_{j}$ and $T_{3}$, then calculates
  (14) $$\begin{array}{c} SK=h(\left(RID_{i}⊕r_{i}\right)\|\left(PSMID_{j}⊕r_{j}\right)),\\ W_{4}=\left(PSMID_{j}\|r_{j}\right)⊕X_{ST}⊕\delta_{j},\\ V_{DE}=h(PSMID_{j}\|r_{j}\|\delta_{j}\left\|T_{3}\right). \end{array}$$
  Finally, $SD_{j}$ sends the message $M_{3}=\left\{W_{4},V_{DE},T_{3}\right\}$ to EGW.
(4) When EGW receives $M_{3}$ sent by $SD_{j}$, first checks $|T_{2}-T_{s}|\leq \Delta T$. If $T_{3}$ is valid, EGW calculates
  (15) $$\begin{array}{c} \left(PSMID_{j}\|r_{j}\right)=W_{4}⊕X_{ST}⊕\delta_{j},\\ V_{DE}=h(PSMID_{j}\|r_{j}\|\delta_{j}\left\|T_{3}\right). \end{array}$$
  Next, EGW checks $V_{DE}^{*}\overset{?}{=}V_{DE}$. If it is correct, EGW authenticates the $SD_{j}$. Next, EGW generates a timestamp $T_{4}$, and calculates
  (16) $$\begin{array}{c} W_{5}=\left(PSMID_{j}\|r_{j}\right)⊕RID_{i}⊕X_{UT},\\ V_{EU}=h(PSMID_{j}\|r_{j}\|RID_{i}\left\|T_{4}\right). \end{array}$$
  At last, EGW sends the message $M_{4}=\left\{W_{5},V_{EU},T_{4}\right\}$ to $U_{i}$.
(5) When receiving $M_{4}$ sent by EGW, $U_{i}$ first checks $|T_{4}-T_{s}|\leq \Delta T$. Then, $U_{i}$ calculates
  (17) $$\begin{array}{c} \left(PSMID_{j}\|r_{j}\right)=W_{5}⊕RID_{i}⊕X_{UT},\\ V_{EU}^{*}=h(PSMID_{j}\|r_{j}\|RID_{i}\left\|T_{4}\right). \end{array}$$
  Finally, $U_{i}$ checks $V_{EU}^{*}\overset{?}{=}V_{EU}$. If the verification is successful, $U_{i}$ calculates
  (18) $$SK=h(\left(RID_{i}⊕r_{i}\right)\|\left(PSMID_{j}⊕r_{j}\right)).$$
  The SK of the $U_{i}$ and $SD_{j}$ is successfully established, indicating the complete login and authentication process.

## 4. Security Analysis

### 4.1. Formal Security Analysis

In this section, we verify the security of the proposed protocol by using the ROR [57,58,59] model. Under the ROR model, different rounds of games are set up to simulate whether an attacker (*A*) can crack the protocol in polynomial time and calculate the SK so as to verify the security of the proposed protocol.

**Adversarial Model.** In this paper, we use commonly used Dolev–Yao [60] and Canetti–Krawczyk [61] models. The following describes the capabilities of *A* in the above model.

(1) *A* can eavesdrop, update, delete, intercept and modify information in the public channel.
(2) *A* can steal the $U_{i}$’s mobile device and then through physical analysis to obtain $U_{i}$’s private information stored in the mobile device [62].
(3) Through a dictionary attack, *A* can guess the $U_{i}$’s identity or password, but *A* cannot simultaneously speculate $U_{i}$’s identity and password.
(4) *A* can obtain the temporary value of any entity.
(5) *A* cannot access information stored in the EGW security database.

**Security Model.** The proposed protocol involves $U_{i}$, EGW, and $SD_{j}$. We define $\Pi_{U_{i}}^{x}$, $\Pi_{EGW}^{y}$, and $\Pi_{SD_{j}}^{z}$ represents the *x*-th $U_{i}$ instance, the *y*-th EGW instance, and the *z*-th $SD_{j}$ instance respectively. Here, assume that the *A* can implement the following operations under the ROR model.

(1) Execute(E): *A* can eavesdrop on messages transmitted between entities, where *E* = {$\Pi_{U_{i}}^{x}$, $\Pi_{GW}^{y}$,$\Pi_{D_{j}}^{z}$}.
(2) $Send(E,M_{i})$: *A* sends the message $M_{i}$ to *E* and get *E*’s response.
(3) Hash(string): *A* enters a string and obtain the string’s hash value.
(4) Corrupt($\Pi_{U_{i}}^{x}$): *A* can get $U_{i}$ information stored in the mobile device.
(5) Test(E): *A* guesses the correct SK by flipping the coin *C*. If *C* = 1, *A* can obtain the correct SK. If *C* = 0, *A* can obtain a random string with the same length as the SK.

According to both models, we adopt Theorem 1 to show the security of our proposed protocol.

**Theorem** **1.** *Under the ROR model, the advantages of the A’s ability to break the proposed protocol in polynomial time ξ are: $Adv_{A}^{P}\left(\xi \right)\leq \frac{q_{h}^{2}}{\left|Hash\right|}+\frac{q_{s}^{2}}{\left|PUF\right|}+2C^{\prime }\cdot q_{send}^{s^{\prime }}$. Here, $q_{h}$ refers to the number of hash operations performed, |Hash| refers to the space of the hash function, |PUF| refers to the PUF function, and $C^{\prime }$ and $s^{\prime }$ refer to two constants.*

**Proof**  We defined five games: $GM_{0}$-$GM_{4}$ to simulate the process of *A* attacking our proposed protocol. In the process of proof, $Succ_{A}^{GM_{i}}\left(\xi \right)$ is defined as the probability of *A* winning in $GM_{i}$, $Adv_{A}^{P}$ is defined as the advantage of *A* to crack the protocol. The specific proof steps are as follows:

$GM_{0}$: In $GM_{0}$, *A* starts the game by tossing a coin *C* and does not perform any operation in the game. Therefore, we can obtain

(19) $$Adv_{A}^{P}\left(\xi \right)=\left|2Pr\left[Succ_{A}^{GM_{0}}\left(\xi \right)\right]-1\right|.$$

$GM_{1}$: By executing Execute(E), *A* can eavesdrop $M_{1}=\{W_{1},W_{2},PID_{i},V_{UE},T_{1}\}$, $M_{2}=\left\{W_{3},V_{ED},T_{2}\right\}$, $M_{3}=\left\{W_{4},V_{DE},T_{3}\right\}$, and $M_{4}=\left\{W_{5},V_{EU},T_{4}\right\}$. When $GM_{1}$ at the end of the session, calculate the SK by executing Test() query, where $SK=h(\left(RID_{i}⊕r_{i}\right)\|\left(PSMID_{j}⊕r_{j}\right))$. However, *A* cannot obtain values $\left\{RID_{i},PSMID_{j},r_{i},r_{j}\right\}$, so *A* cannot calculate SK. Therefore, there is no difference between the probabilities of $GM_{1}$ and $GM_{0}$:

(20) $$Pr\left[Succ_{A}^{GM_{1}}\left(\xi \right)\right]=Pr\left[Succ_{A}^{GM_{0}}\left(\xi \right)\right].$$

$GM_{2}$: Add Send() operation and Hash() operation in $GM_{2}$. Because the authentication values $\left\{V_{UE},V_{ED},V_{DE},V_{EU}\right\}$ are composed of the private value generated by each entity and is secured by the hash function, so *A* cannot tamper with the message. In addition, the random number in the authentication value is different in each session, so a hash collision does not occur. Therefore, according to the birthday paradox, we can obtain

(21) $$|Pr\left[Succ_{A}^{GM_{2}}\left(\xi \right)\right]-Pr\left[Succ_{A}^{GM_{1}}\left(\xi \right)\right]|\leq \frac{q_{h}^{2}}{2|Hash|}.$$

$GM_{3}$: In $GM_{3}$, the difference from $GM_{2}$ is to delete the Hash() operation and add PUF query. As described in Section 1, according to the security attributes of the PUF (·), we can obtain the probability of $GM_{3}$ as

(22) $$\begin{array}{c} |Pr\left[Succ_{A}^{GM_{3}}\left(\xi \right)\right]-Pr\left[Succ_{A}^{GM_{2}}\left(\xi \right)\right]|\leq \frac{q_{s}}{2^{|}PUF|}. \end{array}$$

$GM_{4}$: In $GM_{4}$, *A* obtains the information $\left\{A_{1},R_{1},V_{i}\right\}$ in the mobile device by executing the Corrupt() query, and attempts to exploit the offline password guessing attacks to obtain the user’s correct password $UPW_{i}$. Since *A* cannot obtain the $U_{i}$’s $PID_{i}$ and random number $a_{i}$. The $U_{i}$’s password cannot be guessed. Therefore, according to Zipf’s law [63], we can conclude that

(23) $$|Pr\left[Succ_{A}^{GM_{4}}\left(\xi \right)\right]-Pr\left[Succ_{A}^{GM_{3}}\left(\xi \right)\right]|\leq C^{\prime }\cdot q_{send}^{s^{\prime }}$$

Finally, *A* can only guess bit *C* to obtain the correct SK so as to win the game. Therefore, we can obtain

(24) $$\begin{array}{cc} \frac{Adv_{A}^{P}\left(\xi \right)}{2}\; & =|Pr\left[Succ_{A}^{GM_{0}}\left(\xi \right)\right]-\frac{1}{2}\\ \; & =|Pr\left[Succ_{A}^{GM_{0}}\left(\xi \right)\right]-Pr\left[Succ_{A}^{GM_{4}}\left(\xi \right)\right]|\\ \; & =|Pr\left[Succ_{A}^{GM_{1}}\left(\xi \right)\right]-Pr\left[Succ_{A}^{GM_{4}}\left(\xi \right)\right]|\\ \; & \leq \sum_{i=0}^{3}\left|Pr\left[Succ_{A}^{GM_{i+1}}\left(\xi \right)\right]-Pr\left[Succ_{A}^{GM_{i}}\left(\xi \right)\right]\right|\\ \; & =\frac{q_{h}^{2}}{2|Hash|}+\frac{q_{s}^{2}}{2|PUF|}+C^{\prime }\cdot q_{send}^{s^{\prime }} \end{array}$$

Finally, we can conclude that

(25) $$Adv_{A}^{P}\left(\xi \right)\leq \frac{q_{h}^{2}}{\left|Hash\right|}+\frac{q_{s}^{2}}{\left|PUF\right|}+2C^{\prime }\cdot q_{send}^{s^{\prime }}$$

□

### 4.2. Informal Security Analysis

**MITM Attacks.** It is assumed that *A* can intercept all information transmitted in the public channel. Let us take message $M_{2}$ as an example, message $M_{2}$ contains the authentication value $V_{ED}=h(RID_{i}\|\delta_{j}\|X_{ST}\left\|T_{2}\right)$, *A* tried to tamper with the value of $V_{ED}$, but *A* does not know $RID_{i}$, $\delta_{j}$, and $X_{ST}$, so *A* cannot tamper with the authentication value $V_{ED}$. Similarly, *A* cannot tamper with the message $M_{1}$, $M_{3}$, and $M_{4}$. Therefore, evil intermediaries cannot break our protocol.

**Smart Device Stolen Attacks.** Suppose *A* obtains the information $\left\{S_{2},S_{3},b_{j}\right\}$, which is stored in the memory of $S_{j}$. Since each $S_{j}$ is embedded with a PUF module, *A* is unable obtain the value of $\delta_{j}$ and *A* cannot calculate $PSMID_{j}$. Similarly, *A* cannot calculate $RID_{i}$ and $r_{i}$, so *A* is incapable of successfully calculating SK. Thus, our protocol can resist smart device stolen attacks.

**Temporary Value Disclosure Attacks.** Suppose *A* can obtain the random number generated in any entity. Let us take *A* can obtain $r_{i}$ generated by $U_{i}$ as an example, where $SK=h(\left(RID_{i}⊕r_{i}\right)\|\left(PSMID_{j}⊕r_{j}\right))$. Although *A* can intercept messages in the public channel, *A* cannot know $RID_{i}$, $PSMID_{j}$ and $r_{j}$, so *A* cannot figure out the correct SK. Similarly, even if *A* obtains $r_{j}$ generated by $S_{j}$, it cannot figure out the correct SK. Therefore, even if *A* obtains the random number of any entity, it cannot break our protocol.

**Replay Attacks.** In our proposed protocol, each message delivered in the public channel contains a timestamp. When each entity receives a message, it first checks whether the timestamp is valid. The entity will perform subsequent calculations if the timestamp is within the valid range. Here, take message $M_{2}=\left\{W_{3},V_{ED},T_{2}\right\}$ as an example. Suppose *A* intercepts the message $M_{2}$ and sends $M_{2}$ to $S_{j}$ repeatedly. When $S_{j}$ receiving $M_{2}$ sent by *A*, $S_{j}$ first checks $|T_{2}-T_{s}|\leq \Delta T$. $S_{j}$ will terminate the session because the timestamp in message $M_{2}$ is not within the valid time range. Consequently, our proposed protocol can withstand replay attacks.

**Mutual Authentication.** In our proposed protocol, the validity of the entity is verified by the authentication value. The message passed in the public channel contains the authentication value, wherein

(26) $$\begin{array}{c} V_{UE}=h(RID_{i}\|X_{UT}\|r_{i}\left\|T_{1}\right),\\ V_{ED}=h(RID_{i}\|\delta_{j}\|X_{ST}\left\|T_{2}\right),\\ V_{DE}=h(PSMID_{j}\|r_{j}\|RID_{i}\left\|T_{3}\right),\\ V_{EU}=h(PSMID_{j}\|r_{j}\|RID_{i}\left\|T_{4}\right). \end{array}$$

EGW through calculation of $V_{UE}$ verify the validity of $U_{i}$, $S_{j}$ through calculation of $V_{ED}$ verify the validity of EGW, EGW through calculation of $V_{DE}$ verify the validity of $S_{j}$, $U_{i}$ through calculation of $V_{EU}$ verify the validity of EGW. Therefore, our protocol can ensure that each entity realizes mutual authentication.

**Anonymity and Untraceability.** In our proposed protocol, random numbers and hash functions are used to hide the real identities of $U_{i}$ and $S_{j}$. The pseudonym of $U_{i}$ and $S_{j}$ are used in the authentication process. Even if the attacker intercepts the messages $M_{1}$, $M_{2}$, $M_{3}$ and $M_{4}$ transmitted in the public channel, it cannot track the $U_{i}$ and $S_{j}$. In addition, random numbers are different during each session, ensuring that $U_{i}$ and $S_{j}$ are not traceable. As a result, the proposed protocol can guarantee the anonymity and untraceability of entities.

### 4.3. ProVerif

ProVerif [64,65] is a formal simulation tool developed by Bruno Blanchett for automatically verifying cryptographic protocols. It describes cryptographic primitives, such as hash functions, fuzzy extraction, etc. In this paper, we use ProVerif software to simulate the smart home environment, mainly by executing code to simulate the registration and authentication process of $U_{i}$, EGW, TTP, and $SD_{j}$ to verify the security of our protocol.

**Figure 6:**
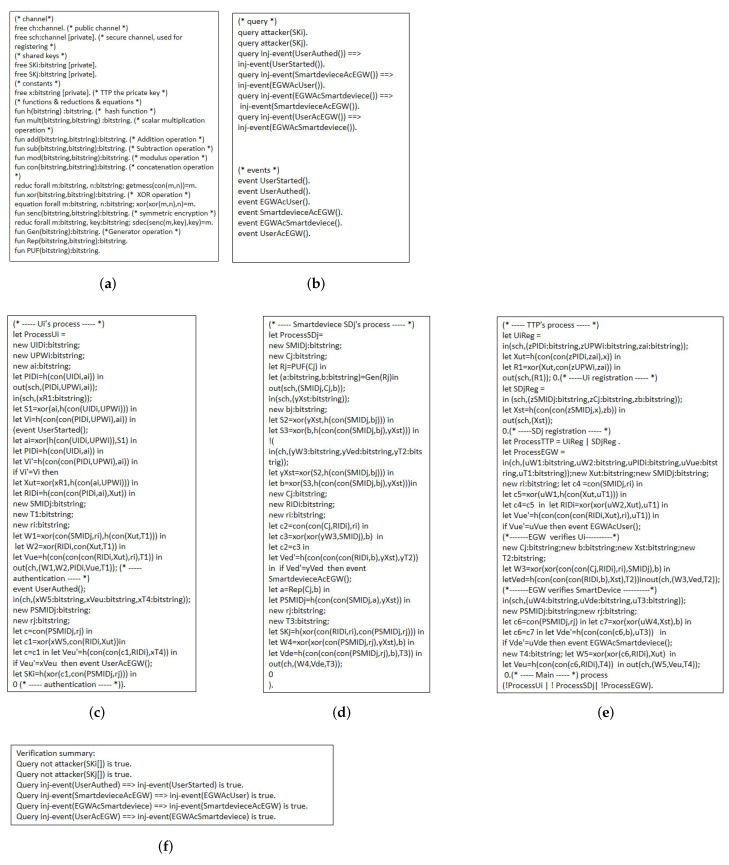
Simulation process in ProVerif. (**a**) Definitions; (**b**) the queries and events; (**c**) execution process of $U_{i}$; (**d**) execution process of $SD_{j}$; (**e**) execution process of TTP and EGW; (**f**) verification results.

The symbols and operations used in ProVerif are defined in Figure 6a. We use ProVerif to query whether *A* can calculate SK through the information transmitted on the public channel. Our proposed protocol proof includes six events: event UserStarted(), event UserAuthed(), event EGWAcUser(), event SmartdeviceAcEGW(), event EGWAcSmartdevice(), and event UserAcEGW(), which indicate that $U_{i}$ starts authentication, $U_{i}$ completes authentication, EGW completes the authentication of the $U_{i}$, $SD_{j}$ completes the authentication of the EGW, EGW completes the authentication of $SD_{j}$, and $U_{i}$ completes the authentication of the EGW. The specific query and event definitions are shown in Figure 6b.

The process of ProVerif simulating $U_{i}$, $SD_{j}$, TTP, and EGW in Figure 6c–e. TTP includes two sub-processes: $U_{i}$ registration and $SD_{j}$ registration. “UiReg” represents the user registration phase, and “SDjReg” represents the smart device registration phase. ProVerif describes the detailed steps of each entity, such as the definition of new parameters and sending and receiving messages. Take the $U_{i}$ process as an example, where “new UIDi: bitstring” represents the definition of the $U_{i}$ identity, “out (sch, (PIDi, UPWi, ai))” represents that the $U_{i}$ sends messages to EGW, and “in (sch, (xR1: bitstring))” means that the $U_{i}$ receives messages sent from EGW. Finally, we use ProVerif to verify the proposed protocol, as shown in Figure 6f. We can conclude from the results that *A* cannot calculate SK, which proves that we propose a secure protocol.

According to the presentations in Section 4.1, Section 4.2 and Section 4.3, we demonstrated the security of our protocol in terms of formal proof (using RoR model), informal proof, and simulation software (ProVerif). The results show that the proposed authentication protocol can resist several well-known attacks, such as insider, gateway impersonation, session key disclosure, offline password guessing, and replay, and provides mutual authentication, anonymity, and untraceability.

## 5. Security and Performance Comparisons

In this section, we compare the proposed protocol with four existing related protocols [18,37,40,42] in terms of security and performance.

### 5.1. Security Comparisons

We compare the security of our proposed protocol with that of Shuai et al. [37], Banerjee et al. [40], Yu et al. [18], and Oh et al. [42]. Table 3 shows the security comparison results. ✓ demonstrates that the protocol can resist this attack, and × demonstrates that the protocol suffers from this attack. Shuai et al.’s protocol [37] suffers from insider attacks, gateway impersonation attacks, session key disclosure attacks, offline password guessing attacks, and replay attacks. Banerjee et al.’s protocol [40] cannot provide anonymity and untraceability. Yu et al.’s protocol [18] is unable to provide mutual authentication. Oh et al. [42] and our protocol can resist these attacks.

### 5.2. Performance Comparisons

We compare the performance from two aspects: computational cost and communication cost.

#### 5.2.1. Computational Cost Comparisons

We compare and analyze the computational costs of each protocol in the login and authentication phase. Additionally, we perform simulation experiments to evaluate the computational cost of the protocol. We use HONOR Play3 to simulate users, Lenovo desktop to simulate edge gateway, and Lenovo laptop to simulate smart devices. The specific configuration of these three devices is shown in Table 4, where the operation time is obtained by averaging 20 times of operation. Here we will ignore hash and join operations. We can see the comparison results of the computational cost from Table 5. Because the running time of the fuzzy extractor is almost the same as that of the hash function, we use the hash function’s running time to represent the fuzzy extractor’s running time in the calculation cost comparison.

**Figure 7:**
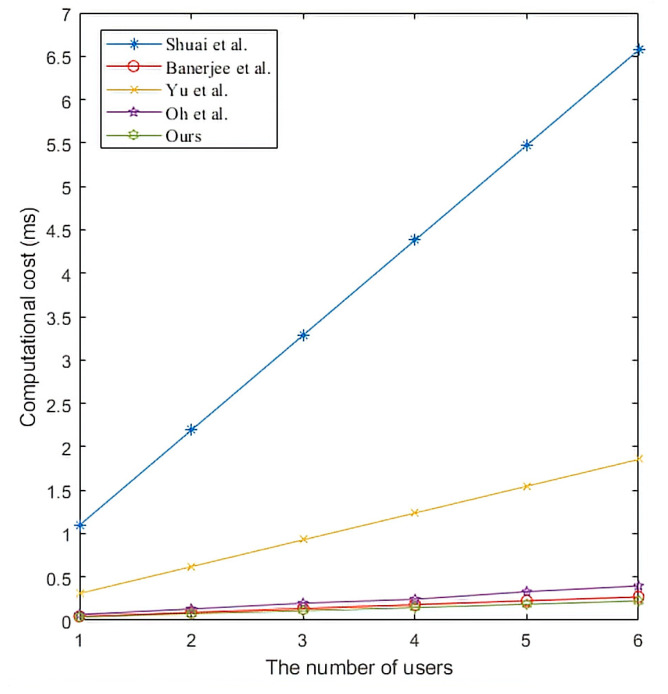
The computational cost of users. Shuai et al. [37], Banerjee et al. [40], Yu et al. [18], and Oh et al. [42].

**Figure 8:**
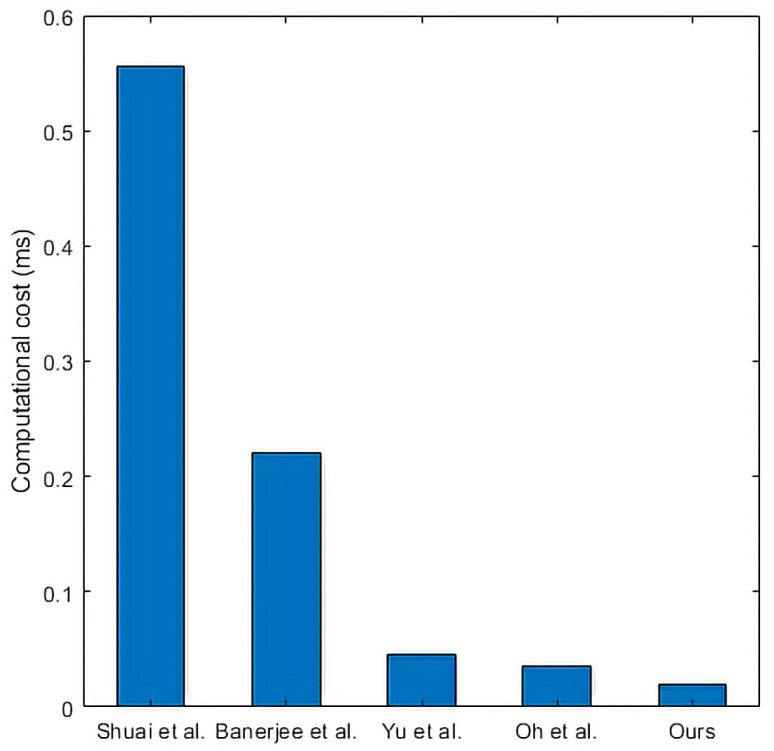
The computational cost of EGW. Shuai et al. [37], Banerjee et al. [40], Yu et al. [18], and Oh et al. [42].

**Figure 9:**
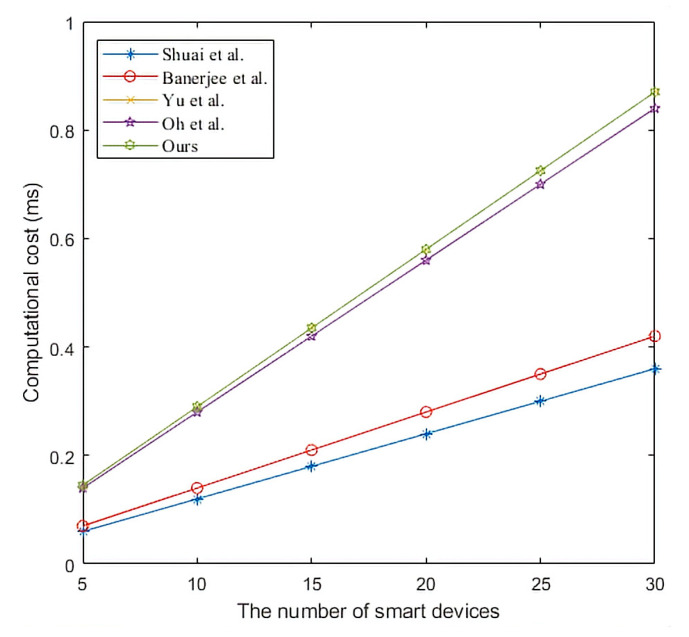
The computational cost of smart devices. Shuai et al. [37], Banerjee et al. [40], Yu et al. [18], and Oh et al. [42].

In the framework of the smart home environment, there can be multiple $U_{i}$ and $SD_{j}$ and only one edge gateway. We describe the relationship between the change in the number of entities and the calculated cost as follows. The relationship between the number of $U_{i}$ and the computational cost is shown in Figure 7. Shuai et al. [37] used point multiplication in the protocol, so the computational cost of this protocol is higher than that of other protocols. Yu et al. [18] used symmetric key encryption/decryption and fuzzy extractor in the protocol, and its computational cost is lower than that of Shuai et al. [37]. Moreover, the computational cost of other protocols is not different. The computational cost of EGW is shown in Figure 8. We can conclude from Figure 8 that the EGW computational cost of the proposed protocol is lower than that of other protocols. The relationship between the number of $SD_{j}$ and the computational cost is shown in Figure 9. We can conclude from Figure 9 that the $SD_{j}$ computational cost of the proposed protocol is lower than that of Oh et al.’s protocol [42], the same as that of Yu et al.’s protocol [18], but slightly higher than that of other protocols.

#### 5.2.2. Communication Cost Comparisons

**Figure 10:**
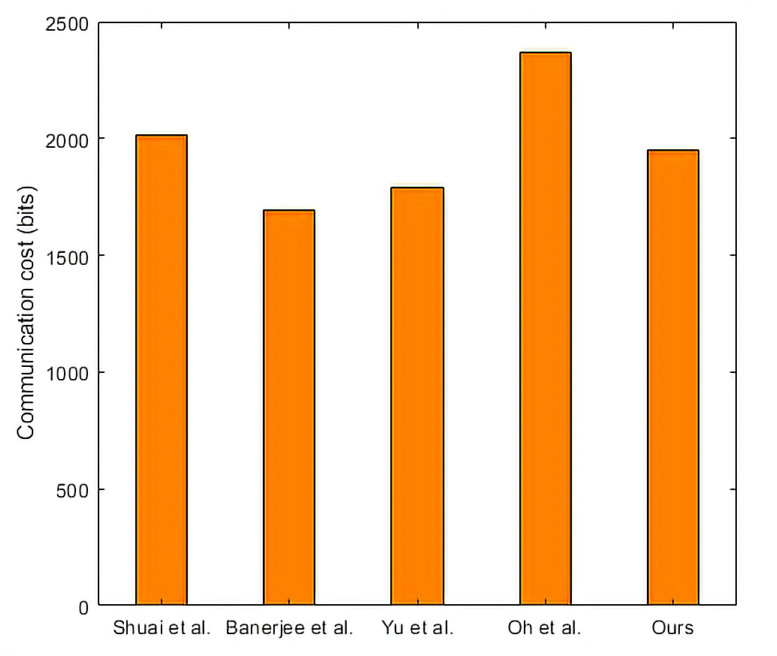
Comparisons of communication cost. Shuai et al. [37], Banerjee et al. [40], Yu et al. [18], and Oh et al. [42].

This part assumes that the length of timestamp, random number, identity, hash function, point multiplication, and symmetric encryption/decryption are 32, 128, 160, 256, 320, and 256 bits. Take our protocol as an example to explain the calculation process of communication cost. In our protocol, the messages transmitted in the public channel are $M_{1}=\left\{W_{1},W_{2},PID_{i},V_{UE},T_{1}\right\}$, $M_{2}=\left\{W_{3},V_{ED},T_{2}\right\}$, $M_{3}=\left\{W_{4},V_{DE},T_{3}\right\}$, $M_{4}=\left\{W_{5},V_{EU},T_{4}\right\}$. Where, $PID_{i}$ is the identity, $\left\{W_{1},W_{2},W_{3},W_{4},W_{5}\right\}$ are random numbers, $\left\{V_{UE},V_{ED},V_{DE},V_{EU}\right\}$ are hash functions, $\left\{T_{1},T_{2},T_{3},T_{4}\right\}$ are time stamps. It is calculated that the communication cost of our protocol is 1952 bits. The communication costs of Shuai et al. [37], Banerjee et al. [40], Yu et al. [18], and Oh et al. [42] are 2016, 1696, 1792, and 2368 bits, respectively. We can draw a conclusion from Table 6 and Figure 10 that the communication cost of the proposed protocol is lower than that of Shuai et al. [37] and Oh et al. [42], and slightly higher than that of Banerjee et al. [40] and Yu et al. [18].

## 6. Conclusions

Communication security is an essential factor for the sustainable development of smart homes. It ensures that users can obtain secure smart home services and protects users’ privacy. Due to the openness of wireless channels prone to data leakage, using cryptographic methods to ensure communication security has attracted many researchers’ attention. To the best of our knowledge, we introduce the first edge-computing-based smart home architecture. Meanwhile, based on this architecture, a PUF-based authentication protocol is proposed. Precisely, the properties of PUF are provided to resist physical tampering and biological cloning attacks. The standard security verification approaches which are formal security analysis using RoR model, informal security analysis, and ProVerif simulation software are made to demonstrate the security of our protocol. The security and performance comparisons are indicated that our protocol has higher security and slightly better performance. In the future, we will adopt several lightweight cryptographic operations to design the new authentication protocol in smart home environments. Without loss of security, the new protocol is more suitable for users’ IoT devices.

## Figures and Tables

**Table 1:** The summary of authentication protocols.

Protocols	Cryptographic Techniques and Properties	Limitations
Yu et al. [18]	(1) Utilized one-way hash function (2) Utilized symmetric encryption	(1) Cannot provide mutual authentication
Jeong et al. [32]	(1) Utilized one-way hash function (2) Based on one-time password	(1) Cannot resist insider attacks (2) Cannot guarantee user anonymity
Vaidya et al. [33]	(1) Utilized one-way hash function (2) Utilized symmetric encryption (3) Utilized HMAC-based one-time password algorithm (4) Based on smart card	(1) Cannot resist provide perfect forward security (2) Cannot resist stolen smart card attacks
Wazid et al. [35]	(1) Utilized one-way hash function (2) Utilized symmetric encryption	(1) Cannot resist stolen verifier attacks (2) Cannot resist synchronization attacks
Shuai et al. [37]	(1) Utilized ECC (2) Utilized one-way hash function (3) Anonymity	(1) Cannot resist insider attacks (2) Cannot resist replay attacks (3) Cannot resist offline password guessing attacks
Kaur and Kumar [38]	(1) Utilized one-way hash function (2) Based on smart card (3) Utilized ECC (4) Two-factor	(1) Cannot resist impersonation attacks (2) Cannot resist ssession key disclosure attacks
Jia et al. [47]	(1) Utilized one-way hash function (2) Utilized ECC (3) Utilize bilinear pairing	(1) Cannot resist MITM attacks
Chen et al. [53]	(1) Utilized one-way hash function (2) Based on PUF (3) Utilized Shamir’s secret sharing	−
Banerjee et al. [40]	(1) Utilized one-way hash function	(1) Cannot guarantee user anonymity and untraceability
Oh et al. [42]	(1) Utilized one-way hash function (2) Based on smart card	−

**Table 2:** Notations.

Notations	Description
$U_{i}$	*i*th user
$SD_{j}$	*j*th smart device
$UID_{i}$	Identity of $U_{i}$
$PID_{i}$	Pseudo-identities of $U_{i}$
$UPW_{i}$	Password of $U_{i}$
TTP	Trusted third party
EGW	Edge gateway
$SMID_{j}$	Identity of $SD_{j}$
*x*	Private key of TTP
Gen(·)	Fuzzy extractor probabilistic generation
Rep(·)	Reproduction function
PUF(·)	PUF function
SK	Session key
h(·)	Secure-hash function
||	Concatenation operation
⊕	XOR operation

**Table 3:** Comparison of security.

Security Properties	[37]	[40]	[18]	[42]	Ours
Insider Attacks	×	✓	✓	✓	✓
Gateway Impersonation Attacks	×	✓	✓	✓	✓
Session Key Disclosure Attacks	×	✓	✓	✓	✓
Offline Password Guessing Attacks	×	✓	✓	✓	✓
Replay Attacks	×	✓	✓	✓	✓
Mutual Authentication	✓	✓	×	✓	✓
Anonymity and untraceability	✓	×	×	×	×

**Table 4:** Configuration parameters and running time of equipment.

	HONOR Play3	Lenovo Desktop	Lenovo Laptop
Operating System	Android System	Windows 10	Windows 10
Running Memory	4G	16G	8G
CPU	HUAWEI Kirin 710F	Intel(R) Core(TM) i5-	Intel(R) Core(TM) i7-
		9500 CPU @ 3.00 GHz	6700HQ CPU @ 2.60 GHz
Hash Function	0.0041 ms	0.0024 ms	0.0035 ms
Point Multiplication	0.5354 ms	0.3354 ms	0.4129 ms
Point Addition	0.1604 ms	0.0633 ms	0.0977 ms

**Table 5:** Computational cost comparison.

Protocols	$U_{i}$ (ms)	EGW (ms)	${\mathrm{SD}}_{j}$ (ms)
Shuai et al. [37]	$2T_{C}+6T_{H}\approx 1.095$	$T_{C}+7T_{H}\approx 0.556$	$3T_{H}\approx 0.012$
Banerjee et al. [40]	$10T_{H}+T_{P}\approx 0.045$	$9T_{H}\approx 0.22$	$4T_{H}\approx 0.014$
Yu et al. [18]	$T_{D}+12T_{H}+T_{P}\approx 0.309$	$11T_{H}\approx 0.045$	$7T_{H}\approx 0.029$
Oh et al. [42]	$16T_{H}\approx 0.066$	$15T_{H}\approx 0.036$	$8T_{H}\approx 0.028$
Our	$9T_{H}\approx 0.037$	$5T_{H}\approx 0.020$	$6T_{H}+T_{P}\approx 0.029$

Here, *T*_*C*_ represents the execution time of ECC point multiplication, *T*_*D*_ represents the execution time of symmetric encryption/decryption operation, *T*_*H*_ represents the running time of hash function, and *T*_*P*_ represents the execution time of the fuzzy extraction function.

**Table 6:** Communication cost comparison.

Protocols	Rounds	Communication Cost
Shuai et al. [37]	4	2016 bits
Banerjee et al. [40]	4	1696 bits
Yu et al. [18]	4	1792 bits
Oh et al. [42]	5	2368 bits
Our	4	1952 bits

## Data Availability

The data is included in the article.

## Abbreviations

The following abbreviations are used in this manuscript:

IoT	Internet of Things
ROR	Real or random
RA	Registration authority
PUF	Physical unclonable functions
AKA	Authentication and key agreement
ECC	Elliptic curve cryptography
